# Evidence-based treatment recommendations for gastroesophageal reflux disease during pregnancy: A review

**DOI:** 10.1097/MD.0000000000030487

**Published:** 2022-09-02

**Authors:** Mansour Altuwaijri

**Affiliations:** a Division of Gastroenterology, Department of Medicine, College of Medicine, King Saud University, Riyadh, Saudi Arabia

**Keywords:** gastroesophageal reflux disease, GERD, heartburn, pregnancy, treatment

## Abstract

Gastroesophageal reflux disease (GERD) occurs in approximately two-thirds of all pregnancies. Around 25% of pregnant women experience heartburn daily. Symptomatic GERD usually presents in the first trimester and progresses throughout pregnancy. The treatment goal is to alleviate heartburn and regurgitation without jeopardizing the pregnancy or its outcome. An English language electronic literature search of MEDLINE, EMBASE, and Cochrane Reviews was undertaken to identify randomized controlled trials, observational studies, management recommendations and reviews of GERD and its treatment during pregnancy. The search period was defined by the date of inception of each database. The treatment in a pregnant GERD patient should follow the step-up approach, starting with lifestyle modification as the first step. If heartburn is severe, medication should be started after consultation with a physician (Recommendation Grade C). The preferred choice of antacids is calcium-containing antacids (Recommendation Grade A). If symptoms persist with antacids Sucralfate can be introduced at a 1g oral tablet, 3 times daily (Recommendation Grade C). Followed by histamine-2 receptor antagonist (Recommendation Grade B). Inadequate control while on histamine-2 receptor antagonist and antacid may mandate a step-up to proton pump inhibitors along with antacids as rescue medication for breakthrough GERD (Recommendation Grade C). This article presented the treatment recommendations for pregnant women with typical GERD, based on the best available evidence.

## 1. Introduction

The concise “Montreal definition,” is used to classify and diagnose gastroesophageal reflux disease (GERD); this defines GERD as a “condition that develops when the reflux of stomach contents causes troublesome symptoms and/or complications.”^[1]^ According to this definition, typical reflux syndrome, a category of the symptomatic esophageal syndromes of GERD that is one of the most common presentations, is characterized by troublesome heartburn, manifesting as a burning sensation in the retrosternal area, and/or regurgitation, perceived as the flow of refluxed gastric contents into the mouth or hypopharynx.^[1]^ Typically, patients themselves determine whether heartburn and regurgitation are troublesome^[1]^; these have been reported as such when mild symptoms are experienced 2 or more days per week, or moderate to severe symptoms are experienced >1 day a week.^[1]^ In practice, clinicians may base treatment decisions on these 2 symptoms alone without performing further diagnostic tests.^[1]^

Despite low disease-related mortality, GERD manifests in poor health-related quality of life, which results in lower physical and mental health, affecting the quality of sleeping, eating, and drinking. Thus, appropriate management strategies are required for immediate control of symptoms and to prevent further deterioration in quality of life, and subsequently, the pregnancy’s well-being.^[2]^

Factors that predispose to pathologic reflux include increasing age, lifestyle, certain beverages and food consumption, overweight, obesity, and pregnancy.^[3–5]^

Pregnant women may experience heartburn daily and with greater frequency as pregnancy progresses.^[4]^ Fortunately, GERD-related complications during pregnancy are uncommon; symptoms are generally limited to the pregnancy period without long-term effects.^[4]^ The management of GERD during pregnancy focuses on relieving heartburn and regurgitation unless it is associated with alarm symptoms (gastrointestinal blood loss, persistent vomiting, dysphagia, chest pain, and involuntary weight loss) which may require further investigation.^[6]^ This article will present the treatment recommendations for pregnant women with typical GERD, based on the best available evidence from Meta-analyses, systematic reviews, randomized clinical trials (RCTs), or good quality observational studies.

### 1.1 Epidemiology

Heartburn occurs in approximately 30% to 50% of pregnancies, reaching 80% in some populations.^[4,7]^ Approximately 17% of pregnant women experience heartburn and regurgitation simultaneously.^[8]^ The incidence of reflux symptoms across the 3 trimesters has recently been reported to be about 25%, with a steady increase in the severity of heartburn over the course of the pregnancy.^[9,10]^

### 1.2 Pathophysiology

The occurrence of GERD during pregnancy is multifactorial, involving both hormonal and mechanical factors. It frequently results from a progressive decrease in lower esophageal sphincter pressure due to incremental increases in circulating estrogen and progesterone.^[11]^ The lowest lower esophageal sphincter pressure occurs at 36 weeks gestation.^[12]^ Other factors that may also play a part in GERD are increased intragastric pressure secondary to the enlarging uterus and changes in gastrointestinal motility through ineffective esophageal motility, with prolonged clearance time.^[11,13]^

## 2. Methods

### 2.1 Source of information

An English language electronic literature search of MEDLINE, EMBASE, and Cochrane Reviews was undertaken to identify randomized controlled trials, observational studies, management recommendations and reviews of GERD, and its treatment during pregnancy. The search period was defined by the date of inception of each database. The earliest was for MEDLINE (1959 through Feb 2022). Search terms included (“gastroesophageal reflux”, “GERD”, “typical reflux syndrome”, “heartburn”, “regurgitation”, “esophagitis”) and (“pregnancy” and “pregnancy trimesters”). Together, the searches yielded 1683 articles. Article observed to relate to the search terms were assessed via titles, abstracts as well as article content. References in relevant articles were also identified and included.

### 2.2 Level of evidence and strength of recommendation

The grading systems used to evaluate the level of the evidence supporting clinical recommendations for the treatment of GERD in pregnancy were adopted from Grading evidence and strength of recommendation guidelines adopted by the American Academy of Family Physicians^[14,15]^. These are summarized in Tables 1 and 2.

**Table 1:** Level of evidence grading.

Level of evidence	Definition
Level A (high quality)	Further research is very unlikely to change our confidence in the estimate of effect.
Level B (moderate quality)	Further research is likely to have an important impact on our confidence in the estimate effect.
Level C (low quality)	There is limited effect in the estimated effect: the true effect maybe substantially different than the estimated effect.
Level D (very low quality)	Any estimate of effect is very uncertain.

**Table 2:** Strength of recommendation grading.

Strength of recommendation	Definition
Strong (A)	Recommendation based on consistent and good quality patient-oriented evidence.
Weak (B)	Recommendation based on inconsistent or limited quality patient-oriented evidence.
Good practice points (C)	Recommendation based on consensus, usual practice, expert opinion, disease-oriented evidence, and case series for studies of diagnosis, treatment, prevention, or screening.

## 3. Treatment and recommendations

The symptoms of heartburn and regurgitation have a sensitivity of 78% and a specificity of 60% to diagnose GERD, and typical GERD can be diagnosed based on these 2 symptoms without additional diagnostic testing.^[6,16]^ However, in the presence of more severe or alarm symptoms or those that might indicate underlying problems, pregnant women should be referred to a gastroenterologist for further investigations.^[17,18]^

**Figure 1.**
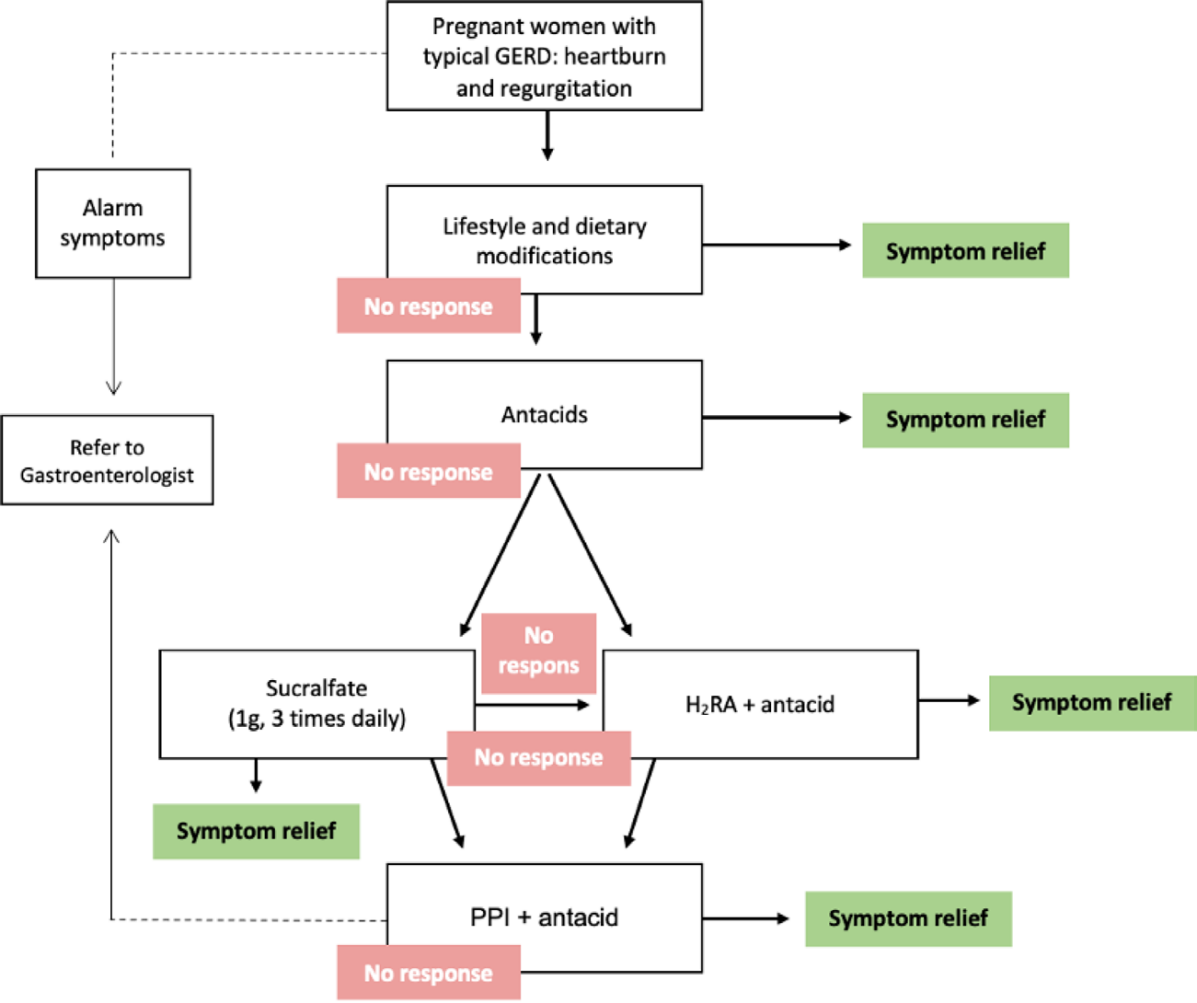
Step-up approach towards management of GERD during pregnancy. GERD = gastroesophageal reflux disease, H2RA = histamine-2 receptor antagonist, PPI = proton pump inhibitor.

Various pharmacological interventions are available for symptom control, but the potential risks to the patient, fetus, and newborn child must be discussed with the patient. The critical teratogenic period during gestation ranges from day 31 (in a 28-day menstrual cycle) to day 71 from the last menstrual period.^[19]^ Exposure to a potential teratogen before this period usually causes an “all-or-none” effect (either fetal death or survival without anomalies); thus, any pharmacological agents that are not absolutely required should be delayed until after the period of potential teratogenicity. Hence, the selected treatment for GERD in pregnancy should minimize the potential risks. Thus, treatment options should follow the step-up approach (Fig. 1; Recommendation Grade C).^[18,20–25]^ In this approach, the first step is lifestyle modification. If there is no response, or troublesome symptoms persist, pharmacological treatment is initiated, beginning with antacids, then histamine-2 receptor antagonists (H2RAs), and, finally, proton pump inhibitors (PPIs) (Table 3).^[20–24]^

**Table 3:** Summary of drugs used to treat typical reflux syndrome during pregnancy.

Drug	FDA classification		Comments
Antacids	None		• Magnesium-, aluminum-, or calcium-containing: most are safe, but calcium-containing drugs are recommended.
			• Magnesium trisilicates: avoid long-term, high dose.
			• Sodium bicarbonate: not safe, may cause fluid overload and alkalosis.
H2RAs	B		• A meta-analysis showed that there is no statistically significant difference in risk of spontaneous abortion, small size for gestational age, or preterm delivery in pregnant women exposed to H2RAs.
PPIs	*Esomeprazole Lansoprazole*	B	• A meta-analysis showed that there is no statistically significant difference in the odds ratios for spontaneous abortion or preterm delivery between pregnant women exposed to PPIs and unexposed pregnant women.
	*Dexlansoprazole*		• A large prospective cohort study found that exposure to PPIs during the first trimester of pregnancy was not associated with a significantly increased risk of major birth defects.
	*Rabeprazole*		
	*Pantoprazole*		
	*Omeprazole*	C	

FDA = Food and Drug Administration, H2RA = histamine-2 receptor antagonist, PPI = proton pump inhibitor.

### 3.1 Lifestyle modifications

Lifestyle and dietary modifications should be considered the first-line treatment in pregnancy, though if heartburn is severe enough, medication should begin after consultation with a physician (Recommendation Grade C).^[4,18,20–27]^

Lifestyle modification measures include: head of bed elevation and lying in the left lateral decubitus position,^[28]^ reducing or avoiding intake of food that may precipitate reflux (e.g., fatty and/or spicy foods, citrus fruits, carbonated beverages, alcohol), and avoiding lying down within 3 hours after eating (Recommendation Grade B).^[29]^

Kaltenbach et al^[28]^ conducted a qualitative, evidence-based, systematic review of 100 articles looking for the efficacy of lifestyle measures in GERD management. They concluded that head of bed elevation and weight loss are effective lifestyle interventions (but the latter would not be used in pregnancy). Head of bed elevation and the left lateral decubitus position reduced esophageal acidity.^[30]^ Furthermore, reducing the intake of fatty food and reducing the size and frequency of meals also suggested to provide better outcomes.^[4,18]^ Obviously, avoidance of smoking, caffeine, and alcohol is also advised. Integrative techniques can also be considered; 1 small RCT study (n = 36) compared the outcomes in heartburn pregnant women who underwent acupuncture vs no acupuncture; women in the acupuncture group were more likely to experience 50% or more improvement in ability to eat and sleep (Evidence Level D).^[31,32]^ The recommended lifestyle and dietary modifications for first-line management of GERD in pregnancy are summarized in Table 4.^[20]^

**Table 4:** Summary of lifestyle modifications for GERD in pregnancy.

Lifestyle modifications for typical reflux syndrome
• Head of bed elevation (6–11 inches) and lying in the left lateral decubitus position.
• Avoidance or reduced intake of food that may precipitate reflux (fatty and spicy foods, citrus, carbonated beverages, and alcohol).
• Avoidance of lying down within 3 hr of eating.

GERD = gastroesophageal reflux disease.

### 3.2 Antacids

If there is no alleviation through lifestyle modifications, pharmacological treatment may be initiated, beginning with antacids. Antacids containing calcium, aluminum, and magnesium are recommended as needed, for second-line treatment of GERD during pregnancy (Recommendation Grade B).^[4,18,20–25]^

If lifestyle measures fail to adequately control symptoms, consensus agreement and experts’ opinion recommend that antacids are used as the initial pharmacological intervention for heartburn in pregnancy and as rescue medication for immediate relief if reflux breaks through with other medications such as H2RAs (Table 3).^[20–24]^

About 30% to 50% of women only require antacids to relieve heartburn during pregnancy and will never step-up from this category.^[11]^ Various types of antacid are available “over-the-counter.” They provide fast and effective, albeit temporary relief of heartburn and are sometimes preferred by patients because they act immediately.

In a moderate-quality RCT of 50 pregnant women with heartburn, 3 different interventions were compared over a period of 7 days. These were magnesium hydroxide plus aluminum hydroxide (antacid) plus oxethazaine, magnesium hydroxide plus aluminum hydroxide without oxethazaine, and placebo. Antacid with or without oxethazaine produced similar relief from heartburn, and increased heartburn relief compared with placebo, with borderline statistical significance (*P* = .05 for either active intervention vs placebo). There were no significant differences in the relief of nausea or regurgitation.^[33]^

In a double-blind RCT of 156 pregnant women suffering from heartburn, an antacid containing a combination of magnesium and aluminum hydroxide with simethicone was compared with placebo. There was a statistically significant difference between the groups: 93% of women reported partial or complete relief of heartburn with the antacid compared to 66% in the placebo group. The antacid in tablets form were significantly superior to placebo (*P* < .01; Evidence Level D).^[34]^

A randomized, double-blind, placebo-controlled, crossover trial of the effects of a low-dose antacid regimen in 47 nonpregnant patients with endoscopically verified reflux esophagitis showed that antacid treatment resulted in lower global symptomatic scores (*P* < .05), fewer days (*P* < .01) and nights (*P* < .05) with heartburn and less acid regurgitation (*P* < .05) than placebo therapy. However, there the method of randomization was not clearly described in this study.^[35]^

A systematic review has been conducted to evaluate interventions for treatment of heartburn in pregnancy. However, the reviewers could not draw solid conclusions about the overall interventions.^[32]^

One retrospective study reported an increased risk of congenital abnormalities^[36]^ in infants exposed to antacids during third trimester of pregnancy; these medications were not teratogenic in animal studies. Antacids have not yet been categorized by the Food and Drug Administration (FDA) (none).^[37]^

The preferred choice of antacids for treating GERD during pregnancy is calcium-containing antacids, in normal therapeutic doses, given the beneficial effect of this treatment in the prevention of hypertension and preeclampsia (Recommendation Grade A).^[20,38,39]^

In a systematic review, calcium supplementation was found to be effective for the prevention of hypertension and preeclampsia.^[38]^ A consensus agreement recommends the use of calcium-containing antacids, given their limited side effects.^[20]^ However, excessive intake of calcium carbonate can lead to the milk-alkali syndrome; calcium carbonate-containing antacids are unlikely to have a significant effect on newborn.^[40]^ Similarly to calcium-containing antacids, magnesium sulfate resulted in 50% reduction in eclampsia risk and, thus, reduced the incidence of maternal mortality in a randomized placebo-controlled trial.^[41]^

It is not recommended to use antacids containing bicarbonate or magnesium trisilicate during pregnancy (Recommendation Grade C).^[42]^

Bicarbonate-containing antacids may cause fetal and maternal fluid overload and metabolic alkalosis.^[42]^ High-dose and prolonged use of magnesium trisilicate is associated with fetal respiratory distress, hypotonia, and nephrolithiasis.^[42]^

#### 3.2.1. Sucralfate.

In patients who persistently exhibiting symptoms of GERD while on antacids, Sucralfate can be the next pharmacological option (1 g oral tablet, 3 times daily) (Recommendation Grade C).^[43]^

Sucralfate is slowly absorbed, giving it the safety to be used in pregnancy and lactation. An animal study demonstrated its safety from teratogenic effects in doses that are 50 times more than those used in human,^[43]^ and it was classified by the FDA as “class B.”^[37]^

Only 1 prospective RCT study evaluated the outcome of this treatment. More women in the Sucralfate group experienced relief of the heartburn and regurgitation when compared to the lifestyle modification group (90% vs 43%, *P* < .05).^[44]^

### 3.3 Acid-suppression therapy

Two types of acid suppressants are used to treat adult GERD; these are H2RAs and PPIs. These have been used widely in pregnancy to treat the discomfort of heartburn and acid indigestion.

#### 3.3.1. Histamine-2 receptor antagonists.

If symptoms persist with antacids alone, H2RAs can be combined with antacids (Recommendation Grade B).^[20,45]^

H2RAs given in combination with antacids, should be considered the third-line treatment of GERD in pregnancy.^[20,45]^

Ranitidine once or twice daily was compared with placebo in a double-blinded, placebo-controlled, triple-crossover study of 18 pregnant women with heartburn who had not responded to antacids or lifestyle modification. The participants continued to receive antacids. Ranitidine (150 mg twice daily) plus antacids significantly reduced symptoms compared to baseline (*P* < .001) and placebo (*P* < .001). Heartburn was reduced by 55.6% (95% confidence interval [CI]: 34.8–76.5) compared with baseline and by 44.2% (95% CI: 15.4–72.9) compared with placebo. No drug reactions or adverse pregnancy outcomes were noted.^[46]^ A consensus has been reached to combine H2RAs with antacids when symptoms persist with antacids alone.^[20]^

The H2RAs are available in several forms, namely cimetidine, famotidine, and nizatidine. In terms of safety, these are all categorized by the US FDA as category B drugs for pregnancy.^[21,37]^ This is because Cimetidine resulted in reduction of testicle and prostate sizes due to its antiandrogenic activity in animal studies. Famotidine was not studied, and ranitidine was not shown to have an antiandrogenic effect on animals, but it was withdrawn from the market in 2020 because of inacceptable levels of N-nitrosodimethylamine.^[47,48]^ A meta-analysis compared 2398 pregnant women exposed to H2RAs in at least the first trimester, with 119,892 unexposed pregnant women; the odds ratio for congenital malformation was 1.14 (95% CI: 0.89–1.45). There was no statistically significant difference in the risk of spontaneous abortion, small size for gestational age, or preterm delivery between exposed and unexposed women (Table 3).^[49]^

#### 3.3.2. Proton pump inhibitors.

If H2RAs combined with antacids cannot sufficiently control the severity of symptoms, it is recommended to use PPIs with the addition of antacids for rescue medication for breakthrough GERD (Recommendation Grade C).^[20,21]^

PPIs provide effective relief from symptoms of reflux among adults. There is no evidence from RCTs or prospective studies about the efficacy of PPIs for control of heartburn or regurgitation in pregnancy. An expert consensus agreement recommends PPIs only for intractable or more troubling symptoms that are not controlled by lifestyle modifications, antacids, or H2RAs with antacids.^[20,21]^ Thus, PPIs represent the fourth-line treatment of GERD in pregnancy.

Available PPI formulations include omeprazole, esomeprazole, lansoprazole, dexlansoprazole, rabeprazole, and pantoprazole. In terms of safety, the FDA categorizes omeprazole as a Class C drug because of potential fetal toxicity (based on evidence from animal studies), while other PPIs are categorized as Class B.^[39]^ In a recent meta-analysis of 1530 pregnant women exposed to PPIs in at least the first trimester compared with 133,410 unexposed pregnant women, the odds ratio for congenital malformation was 1.12 (95% CI 0.86–1.45). There was no statistically significant difference in the risk of major malformations, spontaneous abortion, or preterm delivery.^[50,51]^ Moreover, in a well-designed large prospective cohort study, 5082 pregnant women were exposed to PPIs 4 weeks before conception and toward the end of the first trimester of pregnancy and were compared with 21,811 pregnant women with no PPI exposure. Treatment with PPIs during the first trimester was not associated with a significantly increased risk of major birth defects in a cohort study of 840,000 cases (Table 3).^[52]^

Despite suggested of acid-suppressing agents during pregnancy, a recent meta-analysis showed a statistically significant (*P* < .001) increased risk of asthma in children born to mothers who used PPI or H2RA medications during pregnancy.^[53]^

## 4. Summary and conclusion

Typical GERD in pregnancy can be diagnosed by the characteristic symptoms of heartburn and/or regurgitation without the need for further diagnostic testing. Pregnant women with alarm symptoms or those with severe heartburn or regurgitation should be referred to a gastroenterologist. The following may be used to guide treatment of typical GERD in pregnant women: the treatment plan should follow the step-up approach with lifestyle modification as the first step. If heartburn is severe, medication should begin after consultation with a physician (Recommendation Grade C). Lifestyle modification measures include: head of bed elevation and lying in the left lateral decubitus position, avoidance or reduced intake of food that may precipitate reflux (e.g., fatty or spicy foods, citrus fruits, carbonated beverages, alcohol), and avoidance of lying down within 3 hours of eating (Recommendation Grade B). If there is no alleviation through lifestyle modifications, pharmacological treatment may be initiated, beginning with antacids. Antacids containing calcium, aluminum, and magnesium are recommended as needed as second-line treatment of GERD during pregnancy (Recommendation Grade B). The preferred choice of antacids is calcium-containing antacids, in normal therapeutic doses, given the beneficial effect of this treatment in the prevention of hypertension and preeclampsia (Recommendation Grade A). It is not recommended to use antacids containing bicarbonate or magnesium trisilicate (Recommendation Grade C). If symptoms persist with antacids, a shift to Sucralfate can be reasonable (Recommendation Grade C). If symptoms persist with Sucralfate, an H2RA can be combined with antacids^[20,54]^ (Recommendation Grade B). If H2RAs with antacids provide inadequate control of symptoms, it is recommended to use PPIs along with antacids as rescue medication for breakthrough GERD (Recommendation Grade C). Due to ethical concerns of testing medicine on pregnant women, less advancement is observed in management of GERD in this patient group. Furthermore, quality of available evidence is mostly low (Grade C). Thus, further studies are encouraged.

## Author contributions

Conceptualization: Mansour Altuwaijri.

Data curation: Mansour Altuwaijri.

Formal analysis: Mansour Altuwaijri.

Investigation: Mansour Altuwaijri.

Methodology: Mansour Altuwaijri.

Writing – original draft: Mansour Altuwaijri.

Writing – review & editing: Mansour Altuwaijri.
